# Looking below the surface in plants

**DOI:** 10.7554/eLife.54984

**Published:** 2020-02-11

**Authors:** Rui Wang, Anna A Dobritsa

**Affiliations:** Department of Molecular Genetics, Ohio State UniversityColumbusUnited States

**Keywords:** germline, meiosis, flower, live cell imaging, light sheet microscopy, *A. thaliana*

## Abstract

A new way to culture and image flowers is uncovering the processes that take place in reproductive cells buried deep in plants.

**Related research article** Valuchova S, Mikulkova P, Pecinkova J, Klimova J, Krumnikl M, Bainar P, Heckmann S, Tomancak P, Riha K. 2020. Imaging plant germline differentiation within *Arabidopsis* flowers by light sheet microscopy. *eLife* **9**:e52546. doi: 10.7554/eLife.52546

For cell and developmental biologists, a picture may be worth a thousand words but a movie is priceless. Watching biological processes as they unfold is a powerful way to understand the inner working of organisms: in particular, time-lapse movies can record activity within cells and capture fast events which are easily missed with static snapshots. For example, egg development in female fruit flies had been studied for decades; yet, it is only through advances in culturing, fluorescent labeling and live imaging that scientists realized that eggs acquire their elliptical shape because the chambers that host them during development spin on their axis (Haigo and Bilder, 2011). This, in turn, led to new questions regarding the cellular and molecular mechanisms that contribute to this unusual behavior.

In plant biology, live imaging has already captured the behavior of cells and organs that are naturally exposed on the surface of the plant or can be grown on plates, such as roots and pollen tubes (Hamamura et al., 2014; Prunet et al., 2016; Robinson et al., 2011; Roeder et al., 2010). In these experiments, certain structures inside the cells are labeled with fluorescent proteins, which are then followed using confocal microscopy. This involves shining a laser beam through the entire sample to illuminate and reveal the fluorescence of the tagged proteins, and to collect information about their location. These observations have provided important insights into many genetic and cellular processes, such as gene expression and cell growth, division and differentiation.

In contrast, live imaging has not been widely used to study developmental processes which involve cells – such as male and female germlines – that are hidden deep under layers of opaque plant tissues (Gooh et al., 2015; Sheehan and Pawlowski, 2009). The solution lies in establishing imaging protocols that can expose these cells but also keep them healthy for the duration of the experiments. In 2019, Prusicki et al. successfully grew anthers (the male organs which contain pollen) still attached to young buds in the model plant *Arabidopsis*; using confocal microscopy, they observed the cellular and genetic processes (or meiosis) that lead to the formation of male sexual cells for up to 30 hours (Prusicki et al., 2019). Now, in eLife, Karel Riha of the Central European Institute of Technology at Masaryk University and colleagues in the Czech Republic and Germany – including Sona Valuchova and Pavlina Mikulkova as joint first authors – report a new way to visualize the processes that take place in the reproductive tissues of plants (Valuchova et al., 2020).

The team harnessed a microscopy technique called light sheet fluorescent microscopy (LSFM). In this approach a thin sheet of laser light is used to illuminate the fluorescent proteins in a very small volume of the specimen which is close to the focal plane of the camera on the microscope (Figure 1). This significantly reduces laser damage to the specimen (Ovečka et al., 2018). Detection is often performed perpendicular to the illumination path, with a camera capturing all the signals from the fluorescent proteins in the entire focal plane at once. Compared to conventional confocal microscopy, this design enables higher imaging speeds, and it allows the sample to be rotated and imaged from multiple angles.

**Figure 1.**
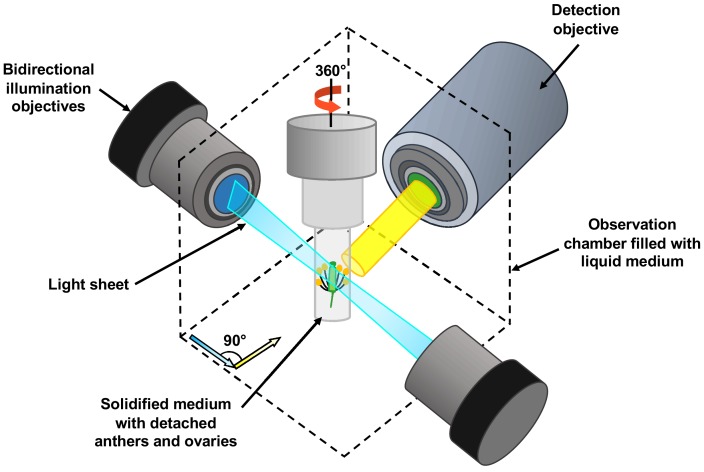
Schematic showing the use of light sheet fluorescent microscopy (LSFM) to image deeply buried reproductive cells in plants. A detached flower bud with sepals and petals removed is submerged in a sugary agarose gel within a sealed capillary (grey cylinder). For long-term imaging, a closed cultivation system was created to allow the detached buds to grow under the microscope without any contamination. Light sheet fluorescent microscopy focuses a thin sheet of laser light (blue) on the specimen: this section overlaps with the focal plane of the detection pathway (in yellow). The light sheet better penetrates the sample, making the imaging of large specimens possible. Only the fluorescent protein tags within the thin sheet of laser light are excited and emit light. This eliminates the out-of-focus excitation and light emission, reducing photodamage in the rest of the sample, and therefore allowing long-term imaging. By moving the sample through the light sheet, the whole volume of the specimen can be imaged plane-by-plane. Samples can also be rotated freely, so the adjustments required by the growth of the specimen can be performed.

Valuchova et al. demonstrate the power of LSFM by following cellular events that take place in the anthers of young *Arabidopsis* buds over the course of several days to produce 4D movies of reasonable spatial resolution. The specimens were prepared by removing the outer floral organs of the buds, and then growing the buds inside small tubes filled with high-sugar and agarose medium (Figure 1). With this approach, the buds could survive for up to five days, and germline development could be observed for longer.

This allowed the team to record the entire process of meiosis in pollen mother cells, as well as the events in their daughter cells and several processes that occur in the tapetum (the surrounding cell layer that nourishes the future pollen). In addition, the spatial resolution of LSFM was sufficient to capture processes inside the cells, such as the movement of individual chromosomes. The technique could also offer high enough temporal resolution to give new insight into these genetic events. For instance, while it is known that pollen mother cells in an anther cavity develop in a synchronized manner, Valuchova et al. noticed that the mother cells at the tip of the anther initiated chromosome segregation slightly later than those at the base. Finally, the team also managed to observe the developing female germline, which is even more challenging.

Seeing is believing, and the approach described in this study, albeit far from simple or readily accessible, opens up exciting opportunities to shed light on other mysteries in plant reproduction, such as how anther and pistil cells differentiate and then communicate, or the intricate exchanges between developing pollen and the tapetum.
